# *In vitro* effects of sodium nitroprusside and leptin on norepinephrine-induced vasoconstriction in human internal mammary artery

**DOI:** 10.5830/CVJA-2014-041

**Published:** 2015

**Authors:** Oktay Burma, Ayhan Uysal, Mete Ozcan, Emine Kacar, Engin Şahna, Ahmet Ayar

**Affiliations:** Department of Cardiovascular Surgery, Faculty of Medicine, Firat University, Elazig, Turkey; Department of Cardiovascular Surgery, Faculty of Medicine, Firat University, Elazig, Turkey; Department of Biophysics, Faculty of Medicine, Firat University, Elazig, Turkey; Department of Physiology, Faculty of Medicine, Firat University, Elazig, Turkey; Department of Pharmacology, Faculty of Medicine, Firat University, Elazig, Turkey; Department of Physiology, Faculty of Medicine, Karadeniz Technical University, Trabzon, Turkey

**Keywords:** internal mammary artery, leptin, contraction, protein kinase C, norepinephrine, sodium nitroprusside

## Abstract

**Aim:**

The biological and pharmacological properties of vessels used in coronary artery bypass graft (CABG) surgery are as important as their mechanical properties. The aim of this study was to investigate the possible role of protein kinase C (PKC)-dependent mechanisms in leptin-induced relaxation in the human internal mammary artery (IMA).

**Methods:**

IMA rings, obtained from patients undergoing CABG surgery, were suspended in isolated tissue baths containing Krebs-Henseleit solution, which were continuously gassed with 95% O_2_ and 5% CO_2_ at 37°C.

**Results:**

The IMA rings were pre-contracted with increasing concentrations of norepinephrine (NE 10^-9^–10^-4^ mol/l) and the relaxation responses to sodium nitroprusside (SNP), a nitrosovasodilator, and leptin were studied in the presence and absence of a PKC inhibitor. Leptin (1 μM) caused a dose-dependent relaxation in NE pre-contracted IMA rings. Pre-treatment with a PKC inhibitor significantly attenuated this vasorelaxatory response to leptin in human isolated IMA.

**Conclusion:**

It was found that SNP and leptin caused significant relaxation of the NE pre-contracted human IMA rings, and PKC was probably the sub-cellular mediator for this effect. Our findings may have clinical or pharmacological importance as it could be hypothesised that obese subjects who have a left IMA bypass graft would have better myocardial perfusion.

## Abstract

Coronary heart disease (CHD) and stroke are the largest contributors to global mortality in low-, middle- and high-income countries as a result of current lifestyles. They will continue to cause decreased quality of life and contribute to the causes of morbidity and mortality throughout the world.^1^,^2^

CHD, also known as coronary artery disease, is the narrowing of coronary arteries, hampering blood and oxygen supply to the heart when plaque builds up in the arteries. The heart is an aerobic organ and disruption of its normal oxygen supply causes irreversible changes in heart tissue. If the disruption of oxygen supply is severe, this becomes life threatening.^3^

Although CHD cannot be cured, there are several treatment options to relieve the symptoms and reduce the progression and risk of complications (heart attack), and thereby prolong the expected lifespan. Treatment options include lifestyle changes and medication, but depending on the severity of the disease, more aggressive treatment methods including interventional procedures (angioplasty and stenting) or coronary artery bypass surgery are warranted.^4^

Revascularisation by coronary artery bypass graft (CABG) surgery is a process of restoring the blood flow around existing blockages to the heart using autologous bypass grafts (or artificial grafts). The immediate success of this procedure is related to surgical technique and the anatomical characteristics of the grafted coronary artery.^5^ After grafting, the vascular smooth muscle cells of the new vessels are the primary regulators of vascular tone. Therefore characterisation of the contractile and relaxatory profiles of the commonly used graft vessels in response to major coronary vasodiladator and vasoconstrictor agents has been carried out in *in vitro* pharmacological investigations.

The effects of noradrenaline, dopamine,^6^ adenosine and nitric oxide^7^,^8^ are well established, but other endogenous agents^9^ that increase in concentration in the circulation during cardiovascular disease are poorly studied. Leptin is a hormone secreted mostly from adipocytes, which is also produced in small amounts from other human tissues such as the heart, stomach, placenta and mammary epithelium.^10^-^14^ In addition to its essential roles in feeding behaviour and energy balance,^11^,^12^ leptin also plays an important role in many different peripheral processes, including haematopoietic, nociception, reproduction, immunity, wound healing, bone remodelling and cognitive functions.^13^

The internal mammary artery (IMA) is the most commonly used vessel in coronary artery grafting to bypass stenosed coronary arteries. Morever its patency rate is longer lasting than the saphenous vein (SV). The IMA has a dynamic vascular bed therefore several vasoactive substances may cause contractile or dilatory responses in the IMA.

Protein kinase C (PKC) is a family of serine/threonine protein kinases. It plays a critical role in the pathogenesis of many heart diseases.^15^-^17^

Although it has been documented that leptin has a vasodilatatory effect,^18^,^19^ the cellular mechanism of this effect is not well documented. The aim of this study was to investigate the possible involvement of PKC-mediated mechanism(s) in the vasorelaxatory effects of sodium nitroprusside (SNP) and leptin on norepinephrine pre-contracted excised human IMA.

## Methods

Informed consent was obtained from the patients and the Clinical Research Ethics Committee of Firat University Medical Faculty (Elazig, Turkey) approved the use of discarded human IMA segments in this study. Segments of the left IMA were collected from 20 patients undergoing CABG. Demographic and clinical characteristics of these patients are given in Table 1.

**Table 1:** Some clinical features of 20 patients undergoing CABG

*Clinical features*	*Mean ± SD, n (%)*
Age	66.5 ± 8.0
Weight	73.2 ± 8.5
Body mass index	27.8 ± 2.6
Gender	
Male	12 (60)
Female	8 (40)
Smoking	9 (45)
Diseases	
Hypertension	17 (85)
Heart failure	3 (15)
Diabetes	10 (50)
Medication	
Organic nitrates	0 (0)
Aspirin	20 (100)
Beta-blockers	14 (70)
Angiotensin inhibitors	9 (45)
Calcium channel blockers	5 (25)
Hypolipidaemics	13 (65)

The IMA were carefully cleaned of loose connective tissue and cut into rings (about 2–3 mm long). The preparations were placed in an isolated tissue bath containing Krebs-Henseleit (KH) solution (composition in mM: NaCl 118, KCl 4.7, MgSO_4_ 1.2, CaCl_2_ 1.25, KH_2_PO_4_ 1.2, NaHCO_3_ 25, glucose 11, EDTA 0.03) at 37°C and pH 7.4, constantly bubbled with a mixture of 95% O_2_ and 5% CO_2_. Contractile activities were recorded using a physiological force transducer (FDT05, Commat Ltd, Ankara, Turkey) recorded by MP150WS for Windows (Biopac Systems Inc, CA, USA).

At the beginning of the experiments, the resting tension of the IMA vessels was adjusted to 1 g and they were allowed to equilibrate under this resting tension for 120 min. Following a stabilisation period, cumulative concentrations of norepinephrine (NE) (10^-9^–10^-4^M) and SNP (10^-9^–10^-4^M) were applied to the organ bath to determine the concentration for a maximum response.

Leptin, NE and SNP were obtained from Sigma (St Louis, MO, USA). The PKC inhibitor, chelerythrine chloride, was obtained from Tocris Bioscience. Each stock solution was diluted to the required concentration immediately before bath application.

## Statistical analysis

Data are presented as mean ± SD. The effects of leptin (1 μM) on contractile activity were evaluated using the unpaired Student’s *t*-test. For all analyses, *p* < 0.05 was regarded as significant.

## Results

The effects of leptin (1μM) and SNP (10-9–10-4M) on the NE concentration (10-9–10-4M) that evoked maximal contractile responses in human IMA rings were studied. The ability of the PKC inhibitor, chelerythrine chloride, to modulate the contractile activity to leptin was also examined.

Firstly we tested the effects of leptin on basal tension. Treatment with leptin (1 μM) did not cause any significant change in basal tension of the IMA rings (data not shown). Cumulative concentrations of NE elicited dose-dependent contraction of the IMA rings. This contractile response was repeatable without any significant run-down (data not shown).

**Fig. 1.**
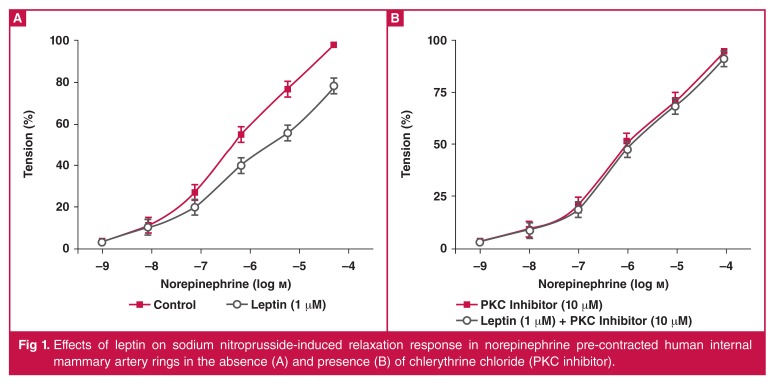
Effects of leptin on sodium nitroprusside-induced relaxation response in norepinephrine pre-contracted human internal mammary artery rings in the absence (A) and presence (B) of chlerythrine chloride (PKC inhibitor).

**Fig. 2.**
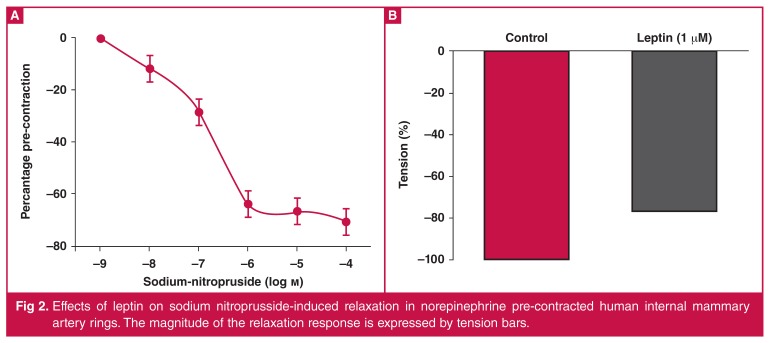
Effects of leptin on sodium nitroprusside-induced relaxation in norepinephrine pre-contracted human internal mammary artery rings. The magnitude of the relaxation response is expressed by tension bars.

In different protocols, the effects of leptin on dose-dependent contractile responses to cumulatively added NE (10^-9^–10^-4^M) were observed. The contractile responses to NE were significantly attenuated by the addition of leptin (1 μM, Fig. 1A, *p* < 0.05, *n* = 20). Cumulatively added SNP-induced vasodilatation (10^-9^–10^-4^M) was also significantly attenuated by leptin (1 μM) (Fig. 2, *p* < 0.05, *n* = 20).

Furthermore, as can be seen in Fig. 1B, 10 μM chelerythrine chloride caused a significant attenuation of vasodilatator response to leptin (Fig. 2, *p* < 0.05). PKC-mediated signalling pathways were probably involved in the leptin-induced vasoactive responses in the human IMA rings.

## Discussion

In the present study, we examined the effects NE, SNP and leptin in isolated human IMA rings. In agreement with the literature and from our clinical results, application of NE to IMA rings caused a dose-dependent contraction. Subsequent application of SNP caused a dose-dependent relaxation. Addition of leptin interrupted the endothelium-independent relaxatory effect of SNP, attenuating its relaxatory effect. Leptin alone did not cause any change in the basal tension of the IMA segments but caused significant relaxation of the NE-induced contractile activity. This is the first study to show that leptin provided a relaxatory effect on the NE-induced contraction of isolated IMA segments, and this effect was PKC dependent.

As presented in Table 1, IMA rings were obtained from a range of patients undergoing CABG surgery due to various cardiovascular diseases. Although most of the associated risk factors have been shown not to affect endothelium-dependent contractile responses of the arteries from these patients,^20^ and both endothelium and smooth muscle are affected by risk factors, we chose to use endothelium-independent relaxation protocols.

The finding of SNP-induced (endothelium-independent) relaxation of the IMA rings indicated that possible injury to the endothelium during harvesting and/or grafting does not totally impair the relaxation capacity of this conduit artery. The contractile functionality of coronary artery grafts has been a topic of substantial interest and has been studied extensively in different vessels, including human saphenous veins, radial artery and IMA.^21^-^24^

Leptin has been shown to cause endothelium-dependent vasorelaxation of the peripheral arteries of experimental animals.^25^ Leptin has also been shown to exert an endothelium-independent vasodilatory action in humans with coronary artery disease.^26^ Therefore, in addition to its central role in the regulation of energy balance and metabolism, leptin has direct effects on the blood vessels (atherogenic, thrombotic and angiogenic) of both coronary and cerebral arteries, potentially contributing to the progression of atherosclerosis in the coronary vessels.^27^-^29^

## Conclusion

By investigating the mechanism and effect of leptin on NE pre-contracted IMA segments, a vessel commonly used for CABG, our *in vitro* study has provided further pharmacological evidence on the characteristics of this vessel. Leptin induced direct vasodilatation of the IMA, and PKC was potentially a sub-cellular mediator for the leptin-induced vasodilatation of these arteries. Although the physiological function of leptin is rather contradictory, as it is associated with left ventricular hypertrophy in hypertensive, insulin-resistant men, it also induces direct vasodilatation through distinct endothelial mechanisms. Our findings therefore may have clinical or pharmacological importance. In the light of these findings, it could be hypothesised that obese subjects who had a left IMA bypass graft would actually have better (anterior wall) myocardial perfusion compared to non-obese subjects. There is a need for further studies investigating possible differences in IMA responses to leptin in vessels from patients with different body mass indexes.
